# Supplementary figures and images for: Identifying publications in questionable journals in the context of performance-based research funding
Source: PLoS One. 2019 Nov 8;14(11):e0224541. doi: 10.1371/journal.pone.0224541 (PMC6839901; doi:10.1371/journal.pone.0224541)

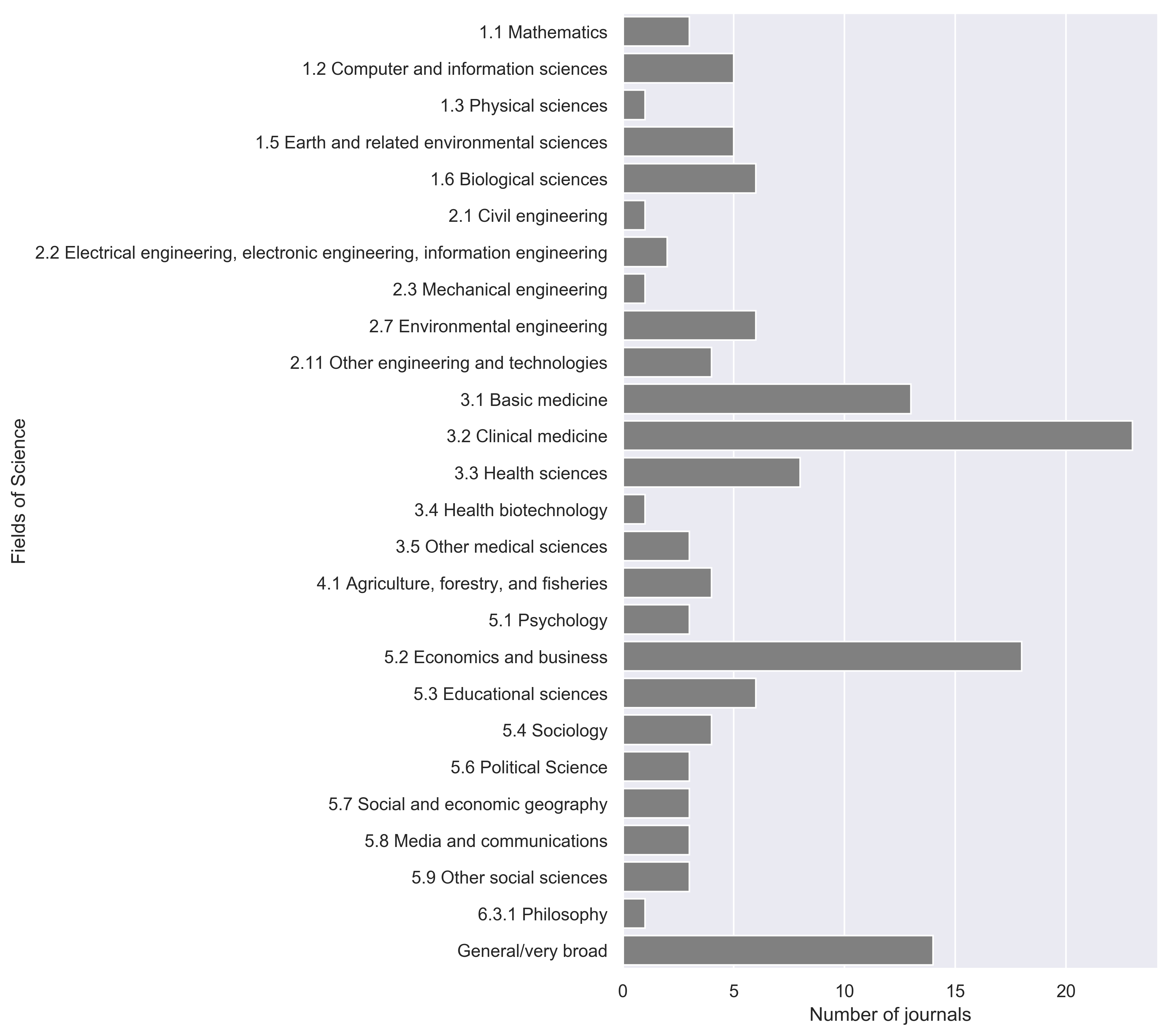

Supplement: S1 Fig — (TIF) [file pone.0224541.s001.tif]
